# Nonlinear and Dotted Defect Detection with CNN for Multi-Vision-Based Mask Inspection

**DOI:** 10.3390/s22228945

**Published:** 2022-11-18

**Authors:** Jimyeong Woo, Heoncheol Lee

**Affiliations:** Department of IT Convergence Engineering, Kumoh National Institute of Technology, Gumi-si 39177, Republic of Korea

**Keywords:** deep learning, mask defect detection, visual inspection system

## Abstract

This paper addresses the problem of nonlinear and dotted defect detection for multi-vision-based mask inspection systems in mask manufacturing lines. As the mask production amounts increased due to the spread of COVID-19 around the world, the mask inspection systems require more efficient defect detection algorithms. However, the traditional computer vision detection algorithms suffer from various types and very small sizes of the nonlinear and dotted defects on masks. This paper proposes a deep learning-based mask defect detection method, which includes a convolutional neural network (CNN) and efficient preprocessing. The proposed method was developed to be applied to real manufacturing systems, and thus all the training and inference processes were conducted with real data produced by real mask manufacturing systems. Experimental results show that the nonlinear and dotted defects were successfully detected by the proposed method, and its performance was higher than the previous method.

## 1. Introduction

**Figure 1:**
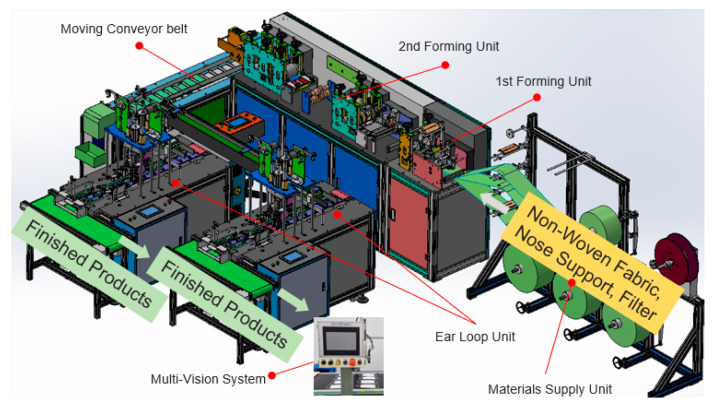
Mask production line with a multi-visual inspection system in mask manufacturing factory.

As COVID-19 spread around the world, the use of masks increased, and subsequently, many automated mask production factories were built. Mask production lines include a fabric supply device for supplying mask fabrics for making mask filters, a mask molding device for molding mask fabrics, and a band attachment device for attaching a band to the mask filters. In the automated production of a mask, after manufacturing a mask by attaching a band to a mask filter, an inspection process is required to inspect the mask for defects before packaging the mask. So, our proposed method algorithm is used in final process with a vision system in a mask manufacturing factory, as shown in Figure 1. In the band attachment test, it is checked whether the band is firmly attached to the mask filter. Things such as dust or hair can be stuck to the mask. These masks must be removed through inspection because they cause discomfort to consumers. During this inspection process, normal and defective products are selected, and defective products are discarded. Human visual inspection is not effective, in terms of fatigue and costs, for determining whether a mask passes or fails inspection. The method introduced in this study strives to resolve these pain points.

Some techniques that are related to image-based inspection systems are Hough transform [1,2,3], local binary patterns (LBP) [4,5], you only look once (YOLO) [6,7], single-shot multibox detector (SSD) [8], RetinaNet [9], scale invariant feature transform (SIFT) [10,11,12], speeded up robust features (SURF) [13,14], artificial neural networks (ANN) [15,16,17], and convolutional neural network (CNN) [18,19,20,21,22]. Hough transform is a technique for detecting straight lines in an image. The detected straight lines have the maximum and minimum values of the x and y coordinates, and various kinds of image processing can be conducted based on these values. LBP is a rotation invariant texture measure that is simple and powerful. YOLO is used for detecting and recognizing various objects in a picture or video. Object detection in YOLO also provides the class probabilities of the detected images. SSD is faster than YOLO. Accuracy is also higher than YOLO. The main idea is predicting category scores and box offsets for a fixed set of default bounding boxes using small convolutional filters applied to feature maps. RetinaNet is composed of a backbone network and two task-specific subnetworks. While having the advantage of the fast detection time of the one-stage detector, the detection performance degradation problem of the one-stage detector was improved. SIFT is an algorithm that extracts features that are invariant to rotation and scale. This method extracts features from two different images and matches the most similar features to find the corresponding parts in the two images. However, the disadvantage of this method is that the processing speed is very low. SURF is an algorithm designed to compensate for the shortcomings of SIFT. SURF uses a method of approximating the LOG with a BOX filter, and the processing speed is made high by adding many characteristic elements in each step of calculating the key point and descriptor. However, when the viewpoint of lighting changes, the features of the image cannot be properly detected. Among these techniques, the CNN and ANN use deep learning and are mainly used for image processing. ANN is an algorithm that mimics the information processing method of the human brain. This technique is used for solving decision-making problems, such as prediction and classification, and it consists of an input layer, a hidden layer, and an output layer. A CNN consists of a convolutional layer, ReLU, and pooling, and when it is given data to learn, it creates a learning model using a fully connected layer. Then, verification is carried out by training and testing the created model. The CNN is useful for finding patterns to recognize images. However, it is hard to find a proper model for a certain application.

The contributions of this paper are as follows:

- The data for training and verification were produced in the real mask production lines.
- An efficient pre-processing process was developed to apply highly small dotted defects, which were difficult to be trained and inferred to the CNN.
- Various types of nonlinear and dotted defects were successfully detected by the proposed method based on the CNN.

The remainder of this paper is organized as follows: Section 2 describes the training problem of the small dotted defect with the masks. Additionally, the reason for using multi-vision-based mask inspection is addressed. In Section 3, preprocessing methods of input data before training and the CNN model are proposed. In Section 4, the evaluation results of the proposed method are shown and compared with other methods quantitatively using a confusion matrix. Finally, Section 5 gives conclusions.

## 2. Problem Description

### 2.1. Multi-Vision-Based Mask Inspection

The visual inspection system uses an automated visual inspection device with a camera. This system can improve efficiency and perform objective and accurate quality control by automating the manual inspection of parts with computer-based camera imaging technology.

**Figure 2:**
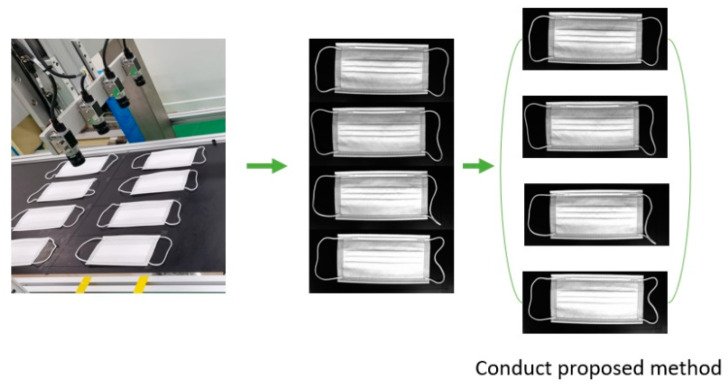
Image processing in multi-vision system.

Several visual inspection systems exist, such as eddy current [23,24,25], thermography [26,27,28], and dye penetrant testing [29,30,31]. Eddy current consists of an electronic sensor and a magnetic coil that induces a magnetic field. The magnetic field is induced in such a way that the interaction between the target magnetic field and the component under examination induces an eddy current that can be measured using an electromagnetic wave sensor. This system has the advantage of being simple and easy, and the inspection can be performed without physical contact. Thermography uses a thermal sensor. A thermal sensor measures the infrared radiation of the inspected component; the radiation flux is converted into a temperature and the temperature distribution is then represented in the form of a thermal image. This system is suitable for surface and interior inspection and has great advantages in detecting large voids or crack defects. Dye penetrant testing detects discontinuities by applying a colored fluid penetrant to the inspection surface. Then, a light source is used by the inspector to highlight the defective features of the surface being inspected. The multi-vision system is a digital camera/software-based system that can visually recognize media registration marks and automatically compensate for distortion, and image drift. In our mask production factory, we use a multi-vision system for this reason, and it improves process time than single-vision system. When taking an image in a multi-vision system, four mask images come in at once. Then, the algorithm divides the four images into quarters and trains each image one by one, as shown in Figure 2, and it is the first algorithm to detect mask inspection using a multi-vision-based system, as shown in Table 1. 

### 2.2. Mask Defect Detection

**Figure 3:**
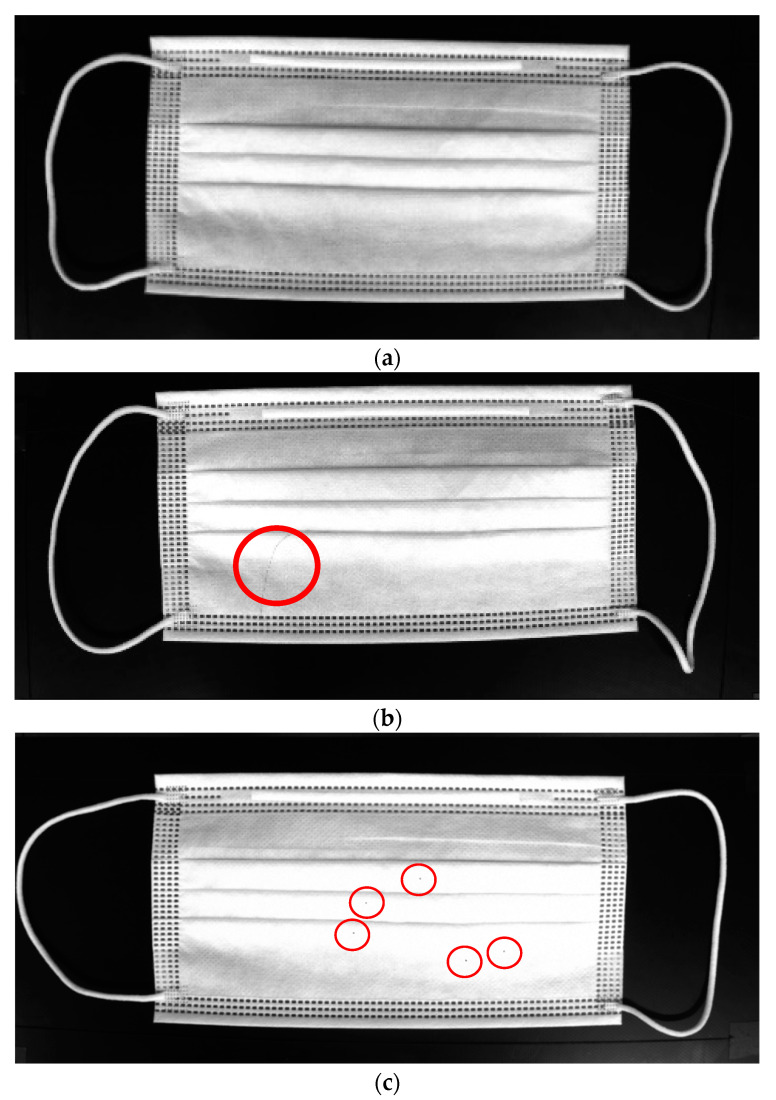

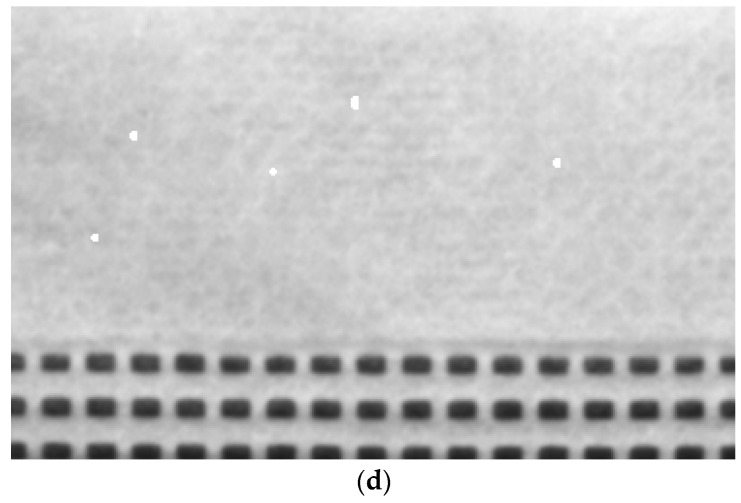
(**a**) Normal mask image; (**b**) nonlinear defect mask image; (**c**) dotted defect mask image; and (**d**) dotted defect mask image.

For a nonlinear defect, which is a thickness of 100 μm attached to a normal mask, as shown in Figure 3b, the mask is classified as a nonlinear defect mask. If a dotted defect is a radius of 100 μm attached to a normal mask, as shown in Figure 3c, the mask is classified as a dotted defect mask. Additionally, white paint or dust may come off during the mask production process, as shown in Figure 3d. It is difficult to distinguish mask defects using a traditional computer image processing method.

Our previous work [32] about detecting nonlinear and dotted defect masks was based on the Pearson correlation coefficient, a calculation that cuts only the filter part of the mask and performs LoG processing to make the defective parts in the filter more visible. After this step, by blurring the average of the mask filter, the difference between the image of the normal mask and the defective area was amplified by changing the pixel values of the part containing the defective part and the surrounding area. The Pearson correlation coefficient was derived by extracting the histogram for each normal and defective mask. This method found the Pearson correlation coefficient per kernel size, and using this value, the minimum value is obtained. Then, the Pearson correlation coefficient value determines whether the mask was normal or defective with that value. It is good to detect different types of nonlinear defect matter in the same mask image, but it is difficult to do this in different mask images because the mask filter patterns may vary slightly, even for a single mask type. However, when we acquired more data from the real vision-based system and tested it, the performance deteriorated. Therefore, we decided to apply the CNN-based method, which is robust to various patterns of defect masks.

## 3. Methods

In an actual automated mask factory, when images are collected with a vision system, four masks are included in one image. Because it is necessary to distinguish between images in the learning process, each image was divided into fourths. With a sufficient amount of data, one-hot encoding was then performed to convert the images into numerical data. After this step, images were classified as normal mask images or defective mask images, and this classification was used to train the model in this study. Finally, the trained model was tested on real images to verify its accuracy.

### 3.1. Data Acquisition

**Figure 4:**
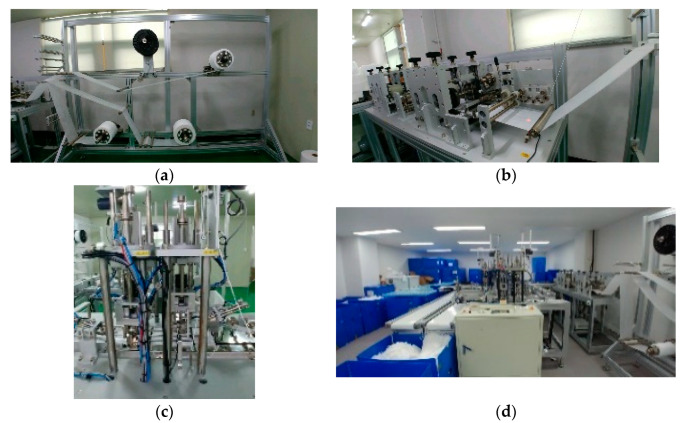
Mask production factory. The non-woven fabric, nose support, and filter are made in a (**a**) materials supply unit. Mask forming is conducted in a (**b**) forming unit. Ear loops are attached in (**c**) ear loop unit 1 and (**d**) ear loop unit 2.

**Figure 5:**
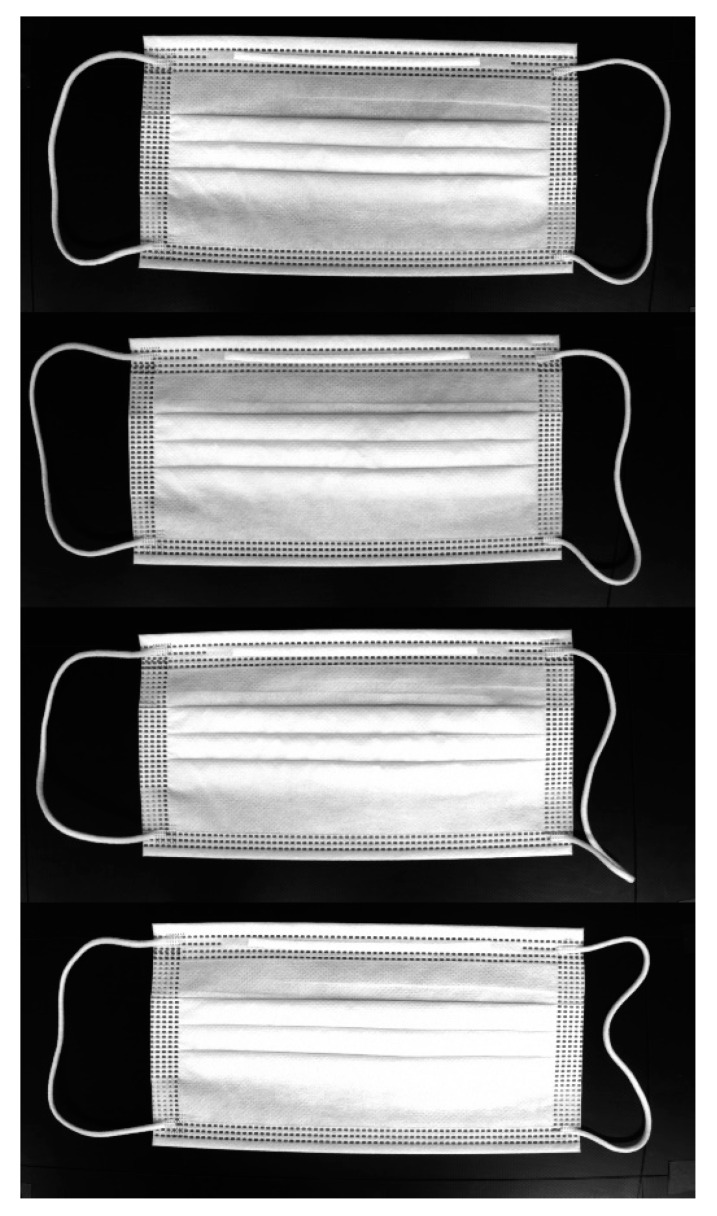
Mask image from a multi-vision system in a mask production line.

Because there were no mask image data that were publicly available, the mask images used in this experiment were produced by a mask manufacturing factory, as shown in Figure 4. After completing all the processes in the mask production line, the mask passes through the multi-vision system, and an image is taken when every 4 masks are produced, as shown in Figure 5. Then, our proposed method is conducted for detecting normal masks and defective masks. In this study, 1000 of the 1300 normal mask images were used for learning, 300 images were used for testing; 1000 of the 1300 defective mask images were used for learning, and 300 were used for testing.

### 3.2. Nonlinear Defect Detection

**Figure 6:**
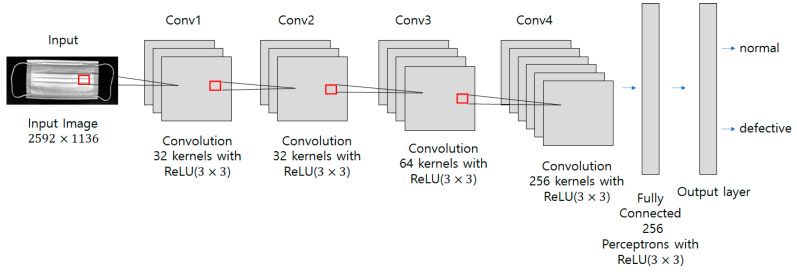
Nonlinear defect CNN model used in the proposed method.

**Figure 7:**
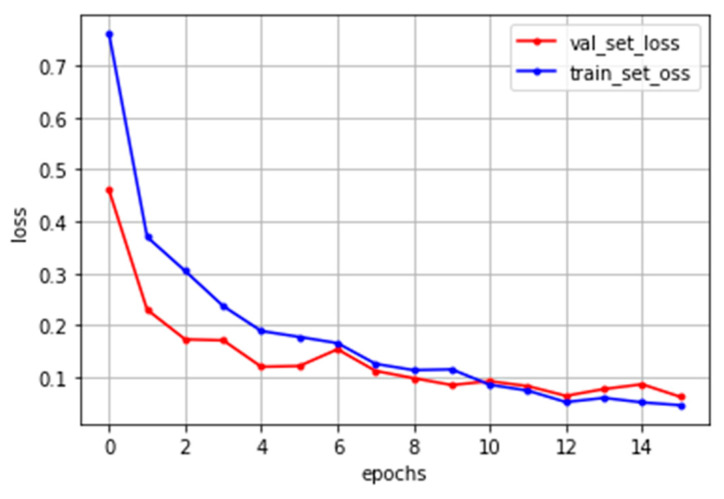
Nonlinear defect CNN model training result.

The deep neural network model proposed in this paper was built as shown in Figure 6. In the nonlinear defect detection CNN model, the input image was 2592 × 1136 pixels, each convolution layer was composed of a 3 × 3 filter, and the padding, which refers to the number of pixels added to an image when it is being processed by the kernel of a CNN, was set to “same”. After each layer trained features in the order of 32, 32, 64, and 256, an activation function, ReLU, was applied, followed by max pooling. The output was then flattened, a fully connected layer with 256 outputs was applied, and the ReLU activation function was applied again. The dense layer was reapplied as many times as the number of output classes. To get the final output, it had a final pass through the softmax layer. When the model was compiled, the loss used binary_crossentropy, which is used for binary classification because there were only two types of data to classify—normal and defective. The Adam optimizer was used, and finally the metrics were set to “accuracy” to find the accuracy. The number of iterations of the model (epochs) was set to 15, as shown in Figure 7, and the batch size was set to 32.

### 3.3. Dotted Defect Detection

In the dotted defect detection preprocessing method, several processes are added. If the entire mask image and background are trained together, it is difficult to train small dots. Therefore, using template matching, crop only the mask filter part for focusing small dots. Template matching is an algorithm that finds an area in the original image that matches the template images. The minimum cross correlation (TM_CCORR) multiplicatively matches the template against the image, so a perfect match will be large and bad matches will be small or 0. The minimum cross coefficient (TM_CCOEFF), which is using the TM_CCORR method after correcting brightness. The minimum square difference (TM_SQDIFF), which is subtracting the pixels of the original image from the template image, squared, and added, is a method of template matching. For the calculation of template matching, the minimum square difference (TM_SQDIFF) is defined as:

(1) $$R\left(x,\;y\right)=\sum_{x^{\prime },y^{\prime }}{\left(T\left(x^{\prime },y^{\prime }\right)-I\left(x+x^{\prime },\;y+y^{\prime }\right)\right)}^{2}.$$

**Figure 8:**
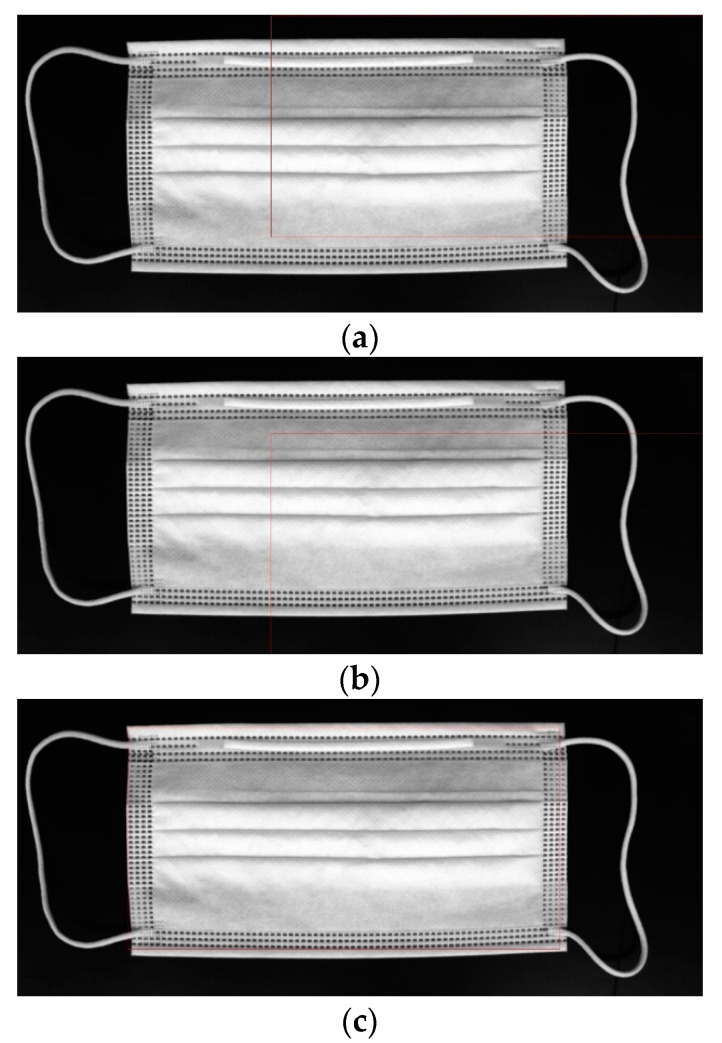
Apply various template matching methods in the mask image, (**a**) apply the minimum cross coefficient method; (**b**) apply the minimum cross correlation method; and (**c**) apply the minimum square difference method.

**Figure 9:**
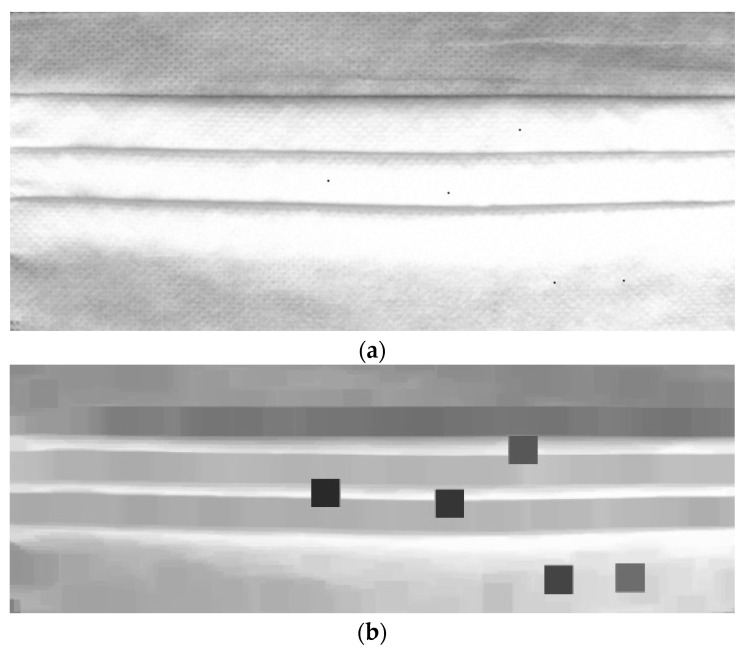
Preprocessing in dotted defect detection. (**a**) Apply template matching to mask image; (**b**) apply morphology erode calculation to (**a**) image.

**Figure 10:**
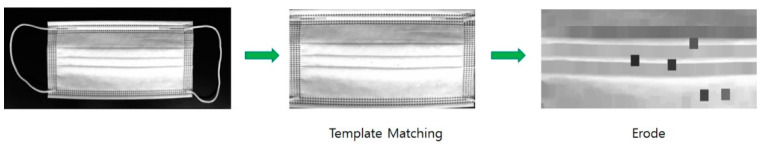
Preprocessing (template matching and erode operation) in dotted defect detection.

**Figure 11:**
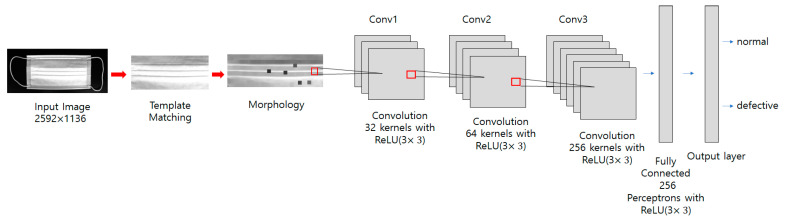
Dotted defect CNN model used in the proposed method.

**Figure 12:**
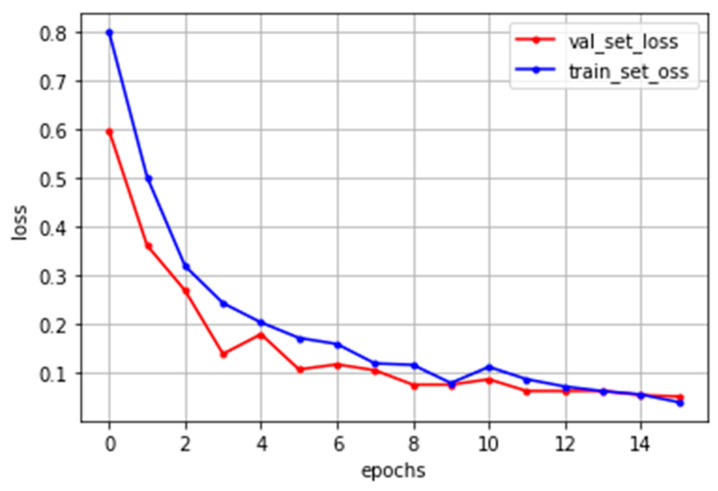
Dotted defect CNN model training result.

In this equation, *x*′ and *y*′ represent the coordinates of the template image. TM_SQDIFF is used for obtaining the difference between the template image and the source image. Place the template image on top of the original image and move it little by little to compare it until reaching the end of the image. The area most similar to the template image is detected in the original image, as shown in Figure 8. After applying template matching, use the morphology erode calculation in OpenCV for making the black dots bigger. The erosion operation replaces the values of all pixels in the kernel area with the local minimum in the kernel. Therefore, applying an erosion operation reduces the bright areas and increases the dark areas, as shown in Figure 9 and Figure 10. However, the morphology erode calculation cannot detect white dotted defective parts. Therefore, our proposed method uses CNN to detect various types of dotted defect masks. The deep neural network model proposed in this paper was built as shown in Figure 11. In the dotted defect detection CNN model, the input image after preprocessing was 2592 × 1136 pixels, each convolution layer was composed of a 3 × 3 filter, and the padding was set to “same”. After each layer trained features in the order of 32, 64, 128, an activation function, ReLU, was applied, followed by max pooling. The output was then flattened, and a fully connected layer with 256 outputs was applied with ReLU. To get the final output, a softmax layer is used. The Adam optimizer was used, and the metrics were set to “accuracy”. The epochs were set to 15, as shown in Figure 12, and the batch size was set to 32.

## 4. Experiments and Results

### 4.1. Mask Defect Detection

**Figure 13:**
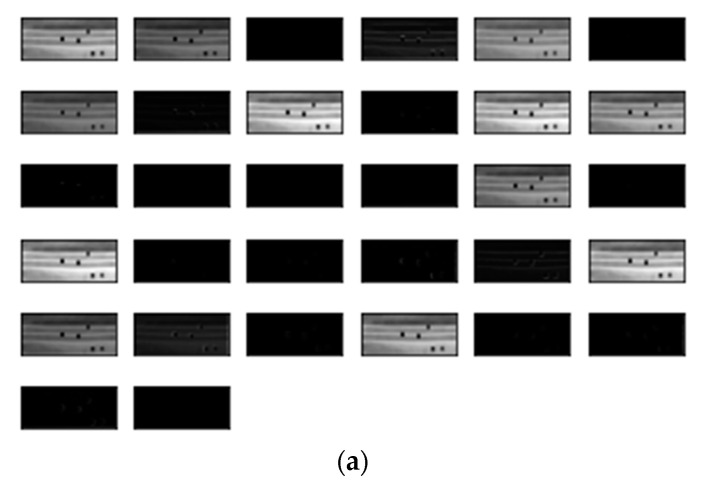

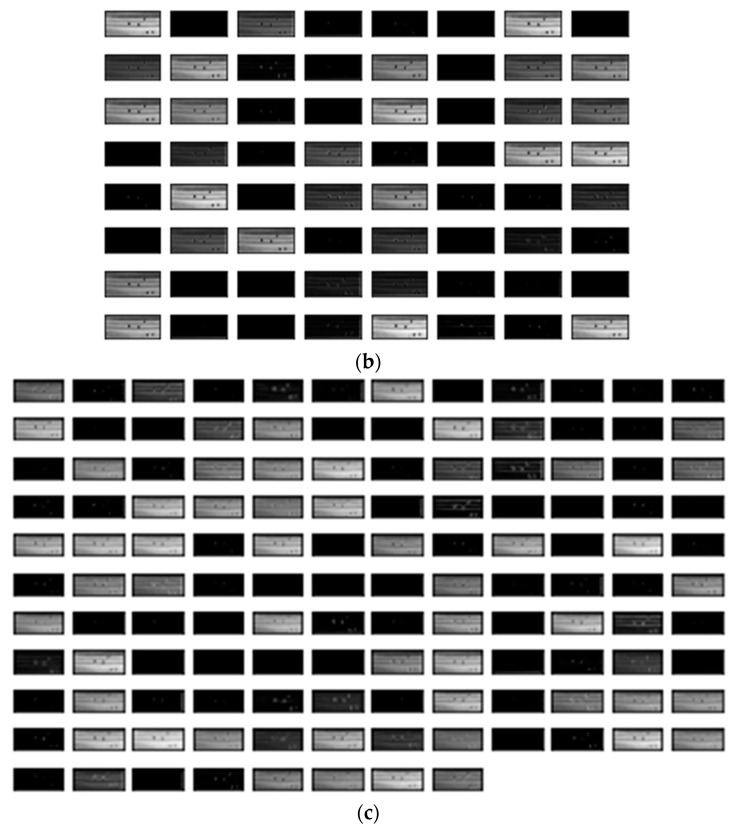
Dotted defect model training results for convolution layers. (**a**) Feature map of the first convolution layer; (**b**) feature map of the second convolution layer; and (**c**) feature map of the third convolution layer.

**Figure 14:**
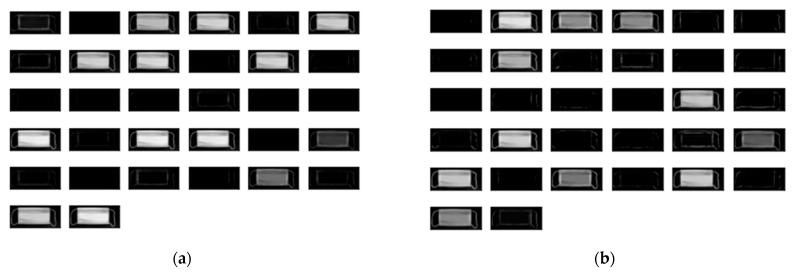

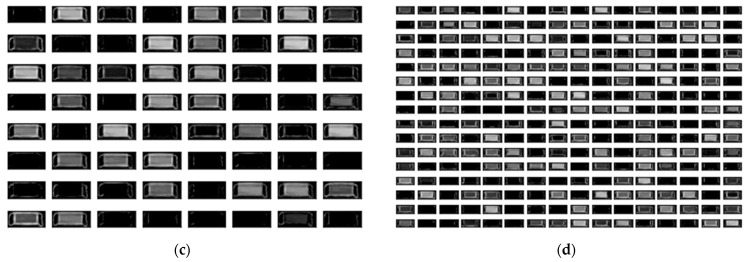
Nonlinear defect model training results for convolution layers. (**a**) Feature map of the first convolution layer; (**b**) feature map of the second convolution layer; (**c**) feature map of the third convolution layer; and (**d**) feature map of the fourth convolution layer.

Our proposed method was conducted on Python 3.7.9, TensorFlow 2.2.0, NVIDIA GeForce RTX 3060. The result of the feature map created by the layers in the neural network proposed in this study is shown in Figure 13 and Figure 14. Table 2 shows the confusion matrix for the mask nonlinear defect detection results. The recall, which is the ratio of the data for which the prediction is true, is 1 for the data having an actual value of true. The precision, where the actual value is the true data ratio, is 0.99 for the data for which the prediction is true. The accuracy, which is an index that evaluates how closely the actual data matches the predicted data, is 99.8%. Computation cost was about 0.06 s per single mask image. However, when we used a single vision-based system, the computation cost was about 0.2 s per single mask image.

Table 3 shows the confusion matrix for mask dotted defect detection results. The recall, which is the ratio of the data for which the prediction is true, is 0.99 for the data having an actual value of true. The precision, where the actual value is the true data ratio, is 1 for the data for which the prediction is true. The accuracy, which is an index that evaluates how closely the actual data matches the predicted data, is 99.8%. Computation cost was about 0.14 s per single mask image. When comparing computation cost in a single-vision-based system and a multi-vision-based system, computation cost was improved form 0.45 s to 0.12 s.

### 4.2. Quantitative Comparison Results 

**Figure 15:**
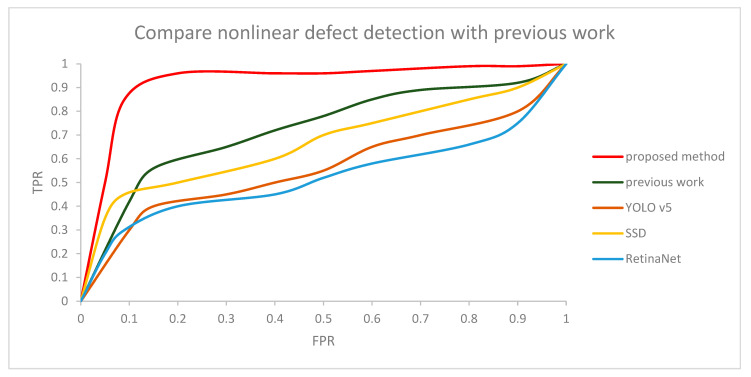
ROC curve for comparing nonlinear defect detection using CNN with previous work.

**Figure 16:**
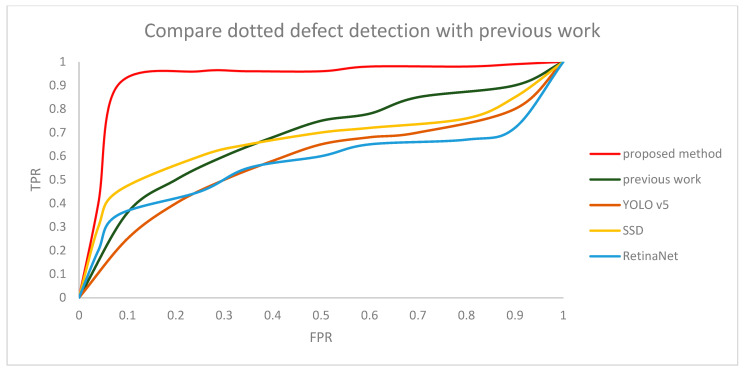
ROC curve for comparing dotted defect detection using CNN with previous work.

A total of 1000 out of 1300 normal images were used for training, and 300 were used for testing. Additionally, 1000 out of 1300 defective images were used for training and 300 were used for testing. Since there is no defect detection algorithm in the actual mask, we compared the proposed method with our previous work [32], SSD, RetinaNet, and YOLO v5. In the proposed method, the threshold for judging normal and defect masks was set to 0.8. Then, we compared the result of the proposed method with previous work according to the change in the threshold, which is shown as a receiver operating characteristic (ROC) curve in Figure 15 and Figure 16. The graph shows our CNN model is robust, even with a changing threshold. The difference in accuracy between the method proposed in this paper, SSD, YOLO v5, RetinaNet, and our previous work is shown in Table 2 and Table 3. The specific model of the YOLO v5 was the YOLO V5l. Computation cost was about 0.3 s per 4 images. Additionally, YOLO v5 had relatively bad results in mask inspection because the line defective part and dotted defective part is too small. So, it is hard to distinguish normal masks and defective masks in YOLO v5. SSD computation cost was about 0.23 s per 4 images. SSD showed better results in mask inspection than YOLO v5, but still the defective part of the mask image was to too small to detect well. RetinaNet computation cost was about 1.2 s per 4 images. Additionally, RetinaNet showed the lowest accuracy compared to other methods. Our previous work computation cost was about 0.4 s per 4 images. Our previous work also shows relatively bad results because when a mask is produced in a factory, it is not always produced in a precise shape, and wrinkles can also occur in various patterns for each mask. The Pearson correlation coefficient method detected with high accuracy only when defective parts were changed in the same image, but when a different mask image was used, the difference between the normal and defective mask values were not big because of the different patterns in each image. On the other hand, when the method proposed in this paper was tested, there was high accuracy, regardless of the different patterns in each image. 

## 5. Conclusions

In this paper, we proposed a suitable CNN structure and proper preprocessing methods for the detection of normal masks and masks having various nonlinear, dotted defects in a mask production line. When the classical computer vision-based method was applied, it was difficult to detect defects due to the diversity of the masks, but the defects were successfully detected when the deep learning-based method with preprocessing was applied. Multi-vision-based system computation time was more improved than the single-vision-based system. The experiment was performed with actual mask image data and showed higher accuracy than other methods for detecting normal masks and defective masks.

## Figures and Tables

**Table 1:** Related works using inspection method.

Related Works	Real Data	Inspection Method	Mask Application
[23,24]	O X	Eddy current	X X
[26,27]	O O	Thermography	X X
[29,30]	O X	Dye penetrant testing	X X
Proposed	O	Multi-vision system	O

**Table 2:** Quantitative comparison results (nonlinear defect detection).

	TP	FN	FP	TN	Recall	Precision	Accuracy
Proposed method	300	0	1	299	1	0.99	99.8%
Previous work [32]	256	44	63	237	0.85	0.8	82.1%
YOLO v5	198	102	88	212	0.66	0.69	68.3%
SSD	223	73	70	230	0.75	0.76	75.5%
RetinaNet	182	118	86	214	0.6	0.67	66%

**Table 3:** Quantitative comparison results (dotted defect detection).

	TP	FN	FP	TN	Recall	Precision	Accuracy
Proposed method	299	1	0	300	0.99	1	99.8%
Previous work [32]	244	56	89	211	0.81	0.73	75.8%
YOLO v5	206	94	119	181	0.68	0.63	64.5%
SSD	229	71	97	203	0.76	0.7	72%
RetinaNet	185	115	141	159	0.61	0.56	57.3%
